# Genetic variability and evolutionary diversification of membrane ABC transporters in plants

**DOI:** 10.1186/s12870-014-0323-2

**Published:** 2015-02-13

**Authors:** Giuseppe Andolfo, Michelina Ruocco, Antimo Di Donato, Luigi Frusciante, Matteo Lorito, Felice Scala, Maria Raffaella Ercolano

**Affiliations:** Department of Agricultural Sciences, University of Naples ‘Federico II’, Via Universita’ 100, 80055 Portici, Italy; CNR – Istituto per la Protezione Sostenibile delle Piante (IPSP-CNR), Portici, Italy

**Keywords:** ATP-binding cassette transporters, Multidrug resistance, *Arabidopsis thaliana*, *Oryza sativa*, *Solanum lycopersicum*, *Solanum tuberosum*, *Vitis vinifera*, *Volvox carteri*, *Saccharomyces cerevisiae*, Gene duplication, Evolutionary dynamics

## Abstract

**Background:**

ATP-binding cassette proteins have been recognized as playing a crucial role in the regulation of growth and resistance processes in all kingdoms of life. They have been deeply studied in vertebrates because of their role in drug resistance, but much less is known about ABC superfamily functions in plants.

**Results:**

Recently released plant genome sequences allowed us to identify 803 ABC transporters in four vascular plants (*Oryza. sativa, Solanum lycopersicum, Solanum tuberosum* and *Vitis vinifera*) and 76 transporters in the green alga *Volvox carteri*, by comparing them with those reannotated in *Arabidopsis thaliana* and the yeast *Saccharomyces cerevisiae*. Retrieved proteins have been phylogenetically analysed to infer orthologous relationships. Most orthologous relationships in the A, D, E and F subfamilies were found, and interesting expansions within the ABCG subfamily were observed and discussed. A high level of purifying selection is acting in the five ABC subfamilies A, B, C, D and E. However, evolutionary rates of recent duplicate genes could influence vascular plant genome diversification. The transcription profiles of ABC genes within tomato organs revealed a broad functional role for some transporters and a more specific activity for others, suggesting the presence of key ABC regulators in tomato.

**Conclusions:**

The findings achieved in this work could contribute to address several biological questions concerning the evolution of the relationship between genomes of different species. Plant ABC protein inventories obtained could be a valuable tool both for basic and applied studies. Indeed, interpolation of the putative role of gene functions can accelerate the discovering of new ABC superfamily members.

**Electronic supplementary material:**

The online version of this article (doi:10.1186/s12870-014-0323-2) contains supplementary material, which is available to authorized users.

## Background

Life is not possible without the exchange of substances and information between cells, therefore macro- and micro-organisms have developed efficient transport systems to control the molecular interaction processes within the colonized environment. Transportation of many molecules through the cell semipermeable membrane against a concentration gradient requires the use of energy that can be provided, for instance, by ATP hydrolysis. Plants, due to their sessile status, have evolved a very complex movement system for molecules, in which proteins belonging to the ABC superfamily play a major role [1].

ATP-binding cassette (ABC) superfamily represents one of the largest protein group within the kingdoms of archaea, eubacteria and eukarya. Proteins belonging to this superfamily are ATP powered transporters able to translocate substrates across cellular membranes. The transported molecules, even if secreted from the same ABC protein, may be extremely different, both in terms of chemistry and structure [2].

The canonical architecture of ABC transporters comprises two transmembrane domains (TMDs) and two cytosolic nucleotide-binding domains (NBDs), also known as ATP-binding cassettes. The structural organisation of the four domains is a dimer of dimers, which can deploy as single polypeptides, or as multisubunit oligomers, reflecting ancient gene duplication events and fusions of the cytosolic catalytic with the membrane-spanning domains [1]. Usually intracytosolic loops are present as extensions of TMDs and function as the interface between the NBDs and TMDs [3]. The NBD contains several highly conserved motifs, including the Walker A and B sequences, the ABC signature motif, the H loop and the Q loop [4].

The ABC signature (alias C motif or LSGGQ motif, ((LIVMFY)S(SG)GX 3(RKA)(LIVMYA)X(LIVFM)(AG)) is situated between the two Walker boxes [5], and is the hallmark that distinguish ABC transporters from other ATP binding proteins.

Due to the complexity and dimension of ABC protein superfamily, a precise classification of all the subfamilies is necessary. The proposed categorisations are various. The transporter classification (TC) system, based on incorporation of both functional and phylogenetic information, includes 53 subfamilies of ABC exporters and 34 of ABC importers (http://www.tcdb.org). Based on sequence comparison, [6,7] three classes of ABC systems, that were probably present in the last common ancestor of archaea, bacteria and eukarya, have been proposed: class 1 comprises transporters with fused TMDs and NBDs (exporters); class 2 includes non-transporter ABCs lacking TMDs; class 3 (which is absent in eukaryotes) includes mainly transporters with NBDs and TMDs formed by separate polypeptide chains (canonical importers), and some bacterial exporters.

Plant genomes encode for a high number of ABC proteins with more than 120 found in both *Arabidopsis thaliana* and *Oryza sativa* [8,9]. The currently used plant ABC protein classification systems are mainly based on phylogenetic information, domain arrangement or similarities/structure comparison with human and microbial prototypes (eg: Pleiotropic Drug Resistance PDR). Sanchez-Fernandez et al. [10] combined information from different classification systems and identified 13 plant subfamilies, including also membrane-bound ABCs that consist only of those containing soluble NBD domains. In order to unify plant and animal ABC naming systems, the Human Genome Organization (HUGO) proposed a new subfamily designation for vertebrate and invertebrate ABC communities, which is now widely used [11,12]. This system originally comprised seven ABC subfamilies (A–G) based on sequence homology, phylogenetic relationships and domain organization. Subsequently, following a more recent inventory of Drosophila and fish ABC proteins, an additional subfamily (H) (not containing members from plants) has been defined. For plants, a further subfamily (I) has been created to incorporate ‘prokaryotic’- type ABCs that are not present in many animal genomes [9]. Subfamilies ABCA, ABCB, ABCC and ABCD contain “forward orientation” TMD-NBD transporters. Subfamilies ABCG and ABCH, instead, are characterized by a “reverse organization” domain NBD-TMD. Subfamilies E and F show only two domains NBD and thus they are labelled as “soluble”. These proteins are not transporters but their NBDs clearly cluster with those of other ABC proteins.

Plant genome sequences availability is growing fast, resulting in an almost completely unexplored repository. The aim of the present work was to list, compare and phylogenetically classify the ABC proteins of selected vascular plant genomes (*A. thaliana*, *O. sativa, Solanum lycopersicum, Solanum tuberosum, Vitis vinifera*), the green alga *Volvox carteri* and the yeast *Saccharomyces cerevisiae*, in order to facilitate future studies on ABC genes and proteins. Identification and classification were based on the work by Verrier and collaborators [9]. Further, a selection pressure study and a customized gene duplication analysis, to identify recent duplication events in vascular plant genomes, have been accomplished. Finally, an expression profile overview of ABC superfamily to detect tissue-specific transporter activation in tomato has been performed as a proof of concept and reported.

## Results and Discussion

### Identification and characterization of putative ABC proteins

A BLASTp search in *O. sativa, S. lycopersicum, S. tuberosum, V. Vinifera* and the green alga *V. carteri* proteomes with Arabidopsis ABC protein dataset allowed us to discover a number of potential ABC sequences. The domain composition of proteins was assessed through a domain detection analysis. A total of 995 proteins (Additional file 1: Table S1) containing one or more ATP-binding cassette domains were identified. Our analysis enlarged number of ABC proteins identified in rice [13], confirming data on ABCG family refined by Matsuda et al. [14], added 32 novel ABC transporters in *V. vinifera*, [15] and provide manual curated ABC protein catalogues for *S. lycopersicum, S. tuberosum.* Most of these proteins belong to known plant subfamilies (A-I, except H) (Additional file 2: Table S2 and Additional file 3). Proteins containing a single domain or novel associations were also recorded. The subfamily A is well conserved among plant species (varying from 6 to 13 members), but it is absent in *S. cerevisiae*. Probably this protein group associated with perturbed cellular lipid transport [8,16], originated after the division of ascomycota and chlorophyta. Subfamily G showed the highest number of members in all the species tested. Subfamilies B and C presented a number of proteins rather stable (ranging from 19 to 38) in the analysed genomes except for *V. carteri* and *S. cerevisiae*. Interestingly, ABC proteins of D, E and F subfamilies represent about 6% of the ABC transporters in *O. sativa, S. lycopersicum, S. tuberosum, V. Vinifera* and *A. thaliana* but about 25% in *V. carteri* and *S. cerevisiae.* The ABCG proteins were found to represent an average of about 40% (ranging from 28% in green alga to 55% in potato) of all annotated transporters in each analysed species. Subfamily G was particularly represented in rice and potato, in which 137 and 93 proteins were annotated, respectively.

**Figure 1:**
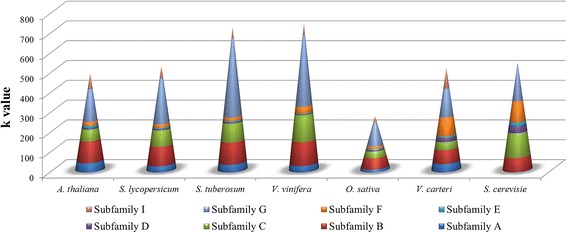
**ABC protein subfamilies profile normalized on proteome size of each analysed species.** k value: ratio between number of ABC protein for each subfamilies (ABCA; ABCB; ABCD; ABCE; ABCF; ABCG and ABCI) and total number of protein sequences for each species.

Normalizing the total number of ABC proteins identified in each species on proteome size, we found a considerable number of ABC proteins in *V. vinifera* and *S. tuberosum* and a much lower number in *O. sativa*. The fraction of single ABC subfamily members in each plant species proteome varies considerably (Figure 1). The *A. thaliana* profile shows an expansion of proteins belonging to subfamily B. *S. tuberosum* and *V. vitifera* has a similar ABC profile with the exception of a slight expansion of subfamily F, whose members have been found involved in growth and development [17]. Also *S. lycopersicum* shows an analogous profile, but in this case, a clear contraction of the proteins belonging to subfamily G has been observed. A triplication event, retained in potato and lost in tomato, contributes to modify the profile of several gene families [18]. Indeed, it has been already demonstrated that tomato and potato genomes differ significantly for R gene complement [19]. Subfamily F is highly represented in *S. cerevisiae* and in *V. carteri*, suggesting an important role in basic processes.

### Selection pressure acting on plant ABC families

The dissimilarity level between the non-synonymous substitution (d_N_) and synonymous substitution (d_S_) values has been used to infer the direction and magnitude of natural selection acting on protein coding genes. In order to discover the selection pressure that characterize the ABC subfamilies in *A. thaliana, O. sativa, S. lycopersicum, S. tuberosum* and *V. vinifera*, we used two different approaches based on Nei-Gojobori and SLAC methods [20,21]. Table 1 shows the results of neutrality tests performed for each ABC subfamilies by using coding DNA sequence (cds) alignments*.* The average δ (d_N_ − d_S_) and ω (dN/dS) value of subfamilies A, B, C, D, and E ranges from −35.31 to −4.54 and from 0.237 to 0.885, respectively, indicating that a negative selection is acting against extreme polymorphic variants. The stabilizing selection that characterizes these subfamilies can be ascribed to the plants need to preserve important protein functions [22,23]. In particular, subfamily E, whose members encode solute-carrier organic anion transporters [24], appeared to be subject to a very strong negative selection pressure (δ = −35.31; ω = 0.237), probably because of its role in RNA degradation [25]. For the ABCF subfamily, the SLAC analysis underlines a soft purification selection (p-Value < 0.05). The subgroup G_WBC_ is the only group that showed a positive average for ω value. Indeed, single codon analysis of the ABCG_WBC_ group underlined 396 positively selected sites (p-Value < 0.05). Finally, the ABCG_PDR_ showed a negative pressure (δ = −13.448 and ω = 0.691), but the single codon analysis underlined 93 codons under positive selection, of which about 15% are located on the first two Pfam NBD ABC_transporter-like domains (PF00005). Probably, the global protein structure of ABCG_PDR_ has been conserved, but positive selection in specific sites of NBD domains has been promoted to generate novel functions [26].

**Table 1:** **Estimation of non synonymous and synonymous substitutions mean dissimilarity for each sub-family (δ = d**_**N**_ **-d**_**S**_ **and ω = d**_**N**_ **/d**_**S**_ **)**

**ABC families**	**n. ABC sequences**	**π**	**Nei-Gojobori method**		**SLAC method**
			**δ**	**p-Value**	**ω**
**A**	49	0.607	−13.61	1e-10	0.656
**B**	131	0.590	−3.56	5e-3	0.911
**C**	95	0.585	−4.54	1e-5	0.768
**D**	10	0.432	−7.47	1e-10	0.885
**E**	10	0.336	−35.31	1e-10	0.237
**F**	28	0.515	0.434	0.664	0.775
**G**_**WBC**_	298	0.710	1.509	0.133	9.624
**G**_**PDR**_	114	0.480	−13.448	1e-10	0.691

In the table are reported: number of ABC sequences analyzed, the average nucleotide diversity (π) of the ABC subfamilies analyzed using the Nei-Gojobori and SLAC methods and the probability (p-Value < 0.05) of rejecting the null hypothesis of strict-neutrality (d_N_ = d_S_;d_N_/d_S_ = 1) in favor of the alternative hypothesis positive selection (d_N_ > d_S_;d_N_/d_S_ > 1) or negative selection(d_N_ < d_S_; d_N_/d_S_ < 1).

### Phylogenetic reconstruction of ABC transporters evolutionary dynamics

**Figure 2:**
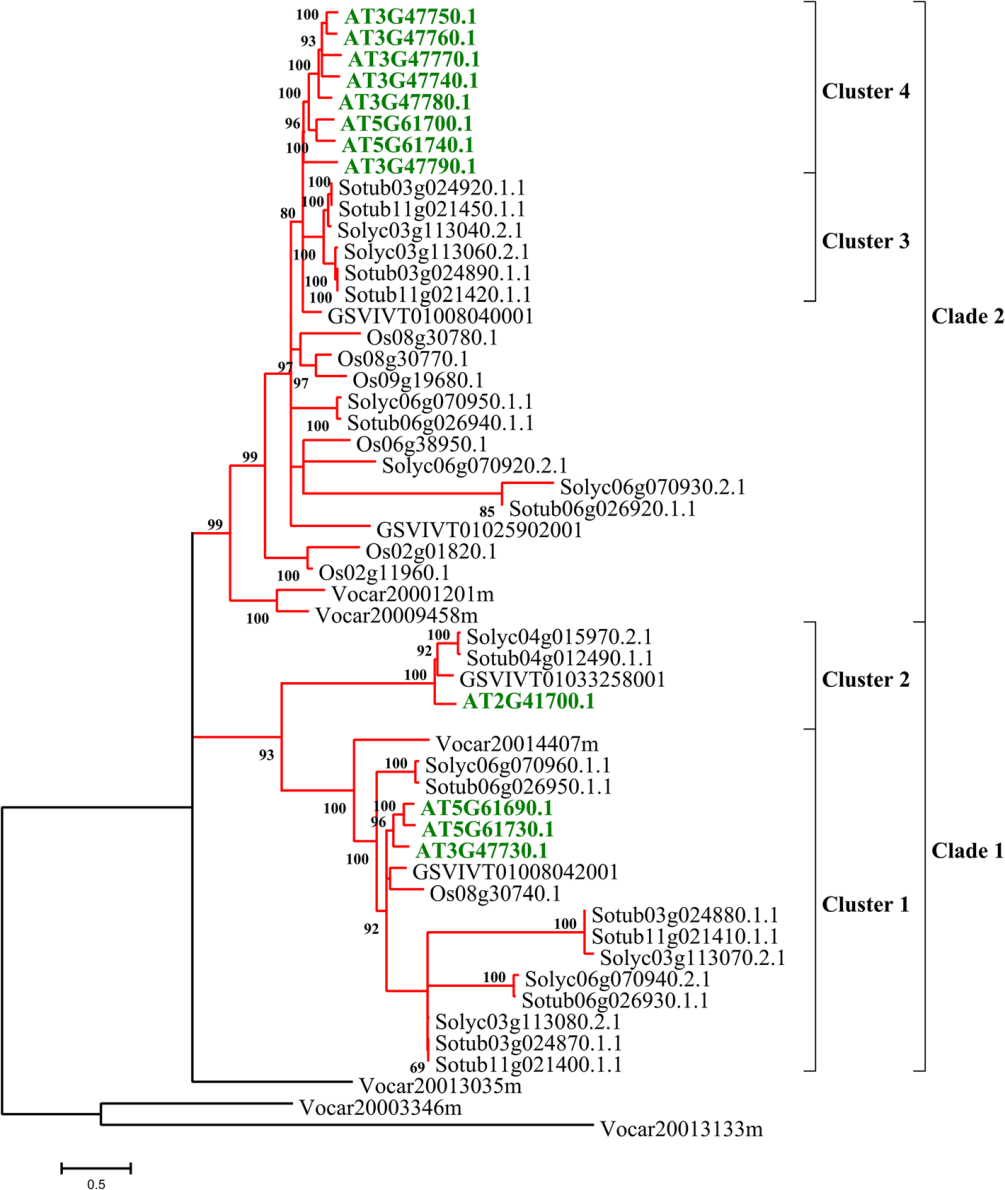
**Phylogenetic tree of ATP-binding cassette subfamily A (ABCA) phylogenetic tree.** The ABCA evolutionary history was inferred using the Maximum Likelihood method based on the Whelan and Goldman model and conducted in MEGA5. Bootstrap values > 70% are indicated above branches. The tree is drawn to scale, with branch lengths proportional to the number of substitutions per site. Identified clades are indicated by numbers and delineated by vertical lines. To facilitate the tree description, the clades were split in “clusters” (subgroups described in more detail).

**Figure 3:**
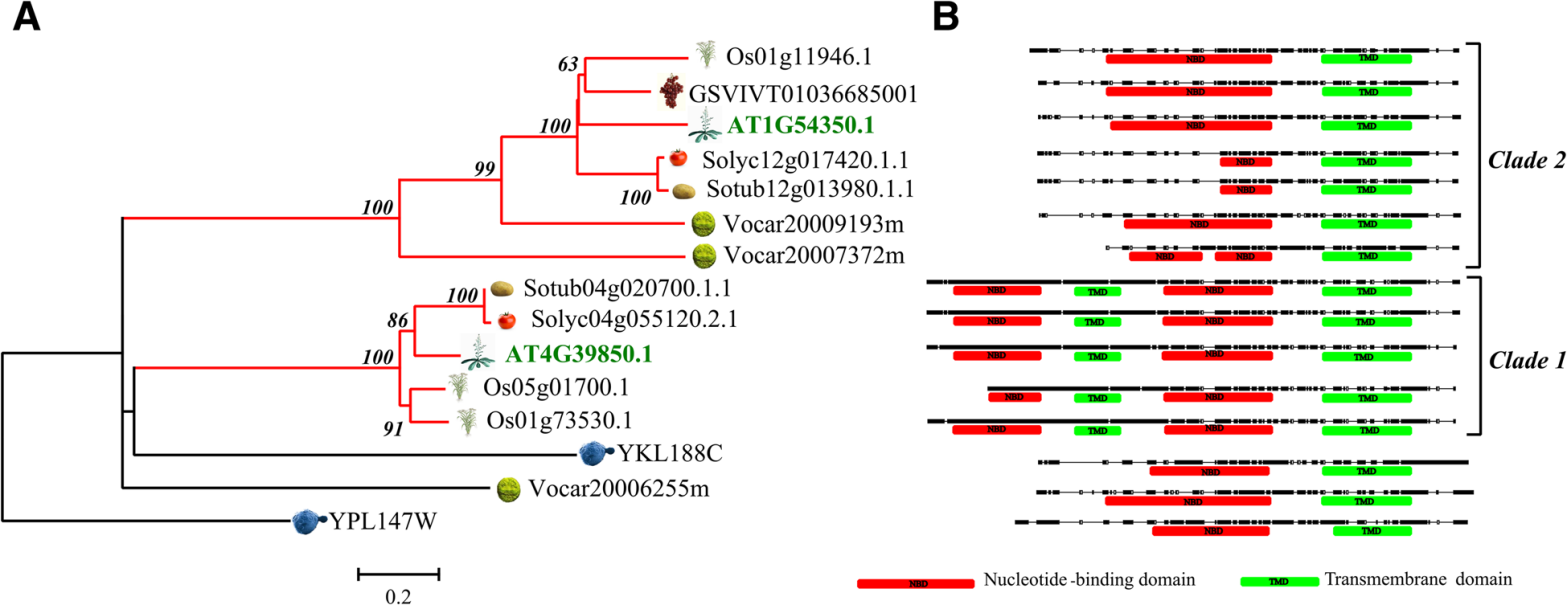
**Phylogenetic tree of ATP-binding cassette subfamily D (ABCD). A**, Evolutionary analyses were performed as reported for ABCA transporter subfamily. **B**, Reconstruction of domain structure of ABCD proteins, refer to PFAM database.

**Figure 4:**
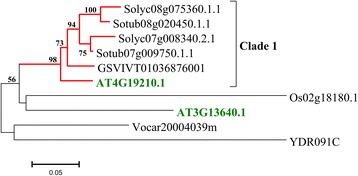
**Phylogenetic tree of ATP-binding cassette subfamily E (ABCE).** Evolutionary analyses were performed as reported for ABCA transporter subfamily.

**Figure 5:**
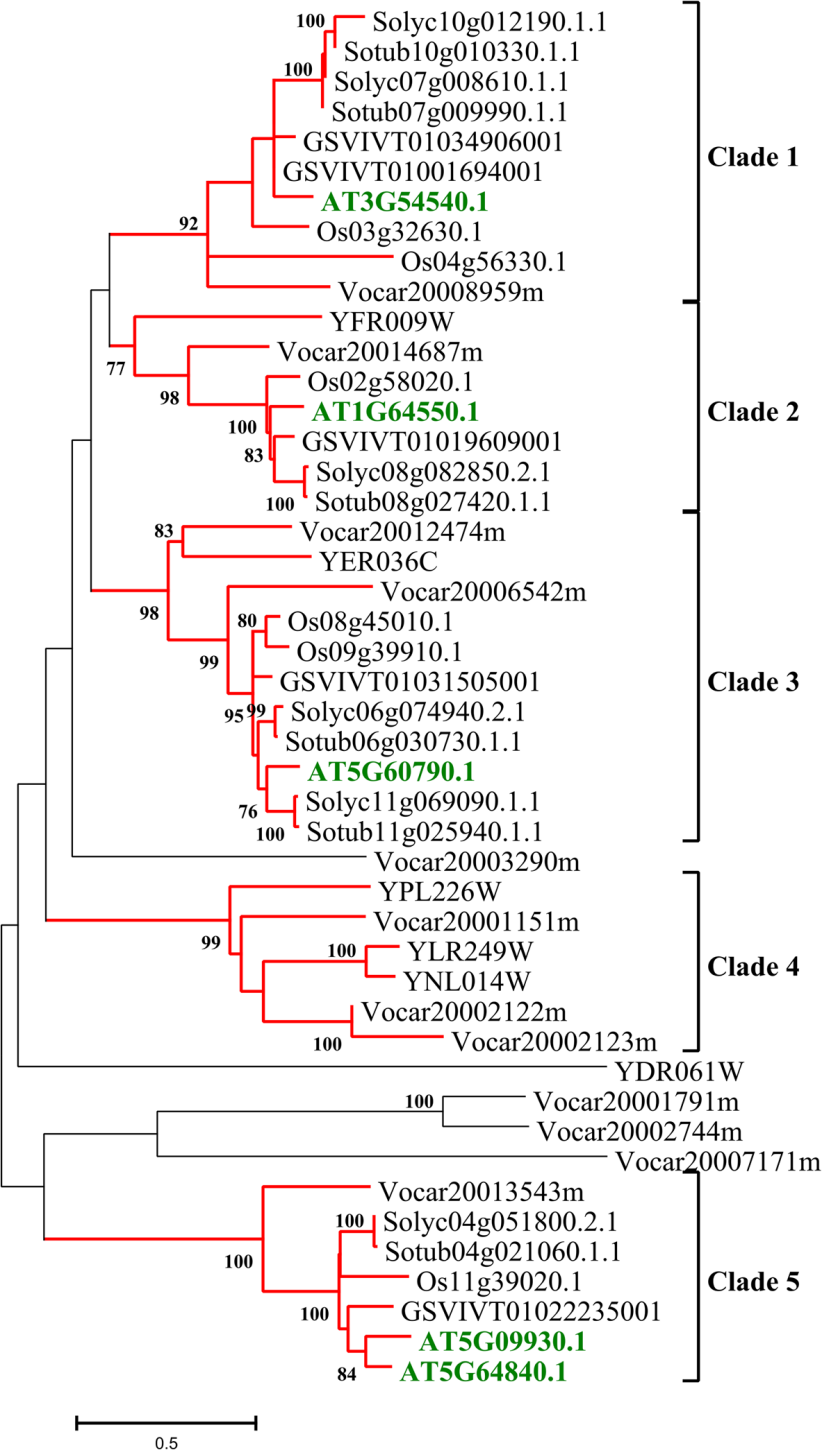
**Phylogenetic tree of ATP-binding cassette subfamily F (ABCF).** Evolutionary analyses were performed as reported for ABCA transporter subfamily.

In order to address questions about evolutionary history of ABC proteins in plants, predicted proteins belonging to subfamily A-G were aligned between them. Furthermore, we performed a maximum likelihood analysis for each ABC subfamily using only complete ABC protein sequences belonging to all the analysed species (Figures 2, 3, 4, 5, Additional file 4: Figure S1 and Additional file 5: Figure S2). The number of proteins analysed for each subfamily varied greatly. The sequences are grouped into robust clades supported by bootstrap values ≥ 70%, while to extract more information from evolutionary histories of each ABC subfamily we highlighted selected subgroups indicated as “clusters”.

#### The ABCA subfamily

ABCA phylogenetic tree (52 protein sequences) shows the presence of three ancestral *V. carteri* sequences, separated from the rest of proteins, and two clades with four clusters with a good bootstrap value (>90) (Figure 2). Clade 1 comprises members belonging to all the species analysed. It seems that the proteins belonging to cluster 1 are well conserved in all species, even if a swift diversification between Arabidopsis and *Solanum spp.* was found. Clade1-cluster 2 shows that a gene expansion occurred only in eudicot genomes. The transmembrane region of four proteins belonging to this cluster (Solyc04g015970.2.1-AT2G41700.1), reveals a string of about 26 amino acids with an alignment identity >70%. Members belonging to this subfamily are involved in cellular lipid transport [8], and a role of full-length ABCA transporter AT2G41700.1, named AtAOH by Sanchez-Fernandez [10], in sterol metabolism has been demonstrated. A similar function for other proteins included in this sub-cluster could be hypothesized. Clade 2 includes 2 ABC transporters annotated in *V. carteri* and 27 in vascular plant proteins, which are subjected to a high degree of differentiation in all angiosperm species with the exception of *V. vinifera*. It is interesting to note that clade 2-cluster 4 contains only 8 *Arabidopsis* proteins with an alignment identity of about 70%, of which six are on chromosome 3, while clade2-cluster 3 contains 6 *Solanum* ABCA proteins with an alignment identity of 90%. A potential translocation of ABC transporters between chromosomes 3 and 11 of potato (Sotub11g021420.1.1-Sotub11g021450.1.1 and Sotub03g024890.1.1-Sotub03g024920.1.1) may have occurred since the two chromosome segments show similar gene arrangements (data not shown).

#### The ABCB subfamily

The ABCB evolutionary history (Additional file 4: Figure S1) was inferred by analysing 169 proteins. Several sub-groups were identified in this subfamily, suggesting a large diversification among the analysed species. The phylogenetic tree displayed 13 main clades, supported from high internal branch bootstrap indexes. Subsequently, modifications of original sequence arrangement produced few sequences in yeast and algae, and a huge number in vascular plants. Phylogenetic analysis suggests that sub-groups evolved differently in each species. However, orthologous of six Arabidopsis proteins present in clade 1 were identified both in monocot and dicot species. Proteins belonging to this subfamily seem to be involved in auxin influx transport in roots, and contribute to the basipetal transport in hypocotyls and root tips by establishing an auxin uptake sink in the root cap. Moreover, they confer sensitivity to 1-N-naphthylphthalamic acid (NPA), regulate root elongation, initiation of lateral roots and development of root hairs, transport IAA, indole-3-propionic acid, NPA syringic acid, vanillic acid and some auxin metabolites, but not 2,4-D and 1-naphthaleneacetic acid [27,28]. In particular, AT2G36910.1 and AT3G28860.1 are involved in auxin transport in stems and root, respectively [29,30]. It is possible to predict similar functions for orthologous proteins and gain insight in species not yet characterized by looking at specific clade arrangements. For instance, clade 8 embraces ten transporters afferent to all the species analysed. *S. cerevisiae* (YMR301C) and Arabidopsis (AT4G28620.1, AT4G28630.1 and AT5G58270.1) were found to be involved in iron homeostasis [31] suggesting that this function is well conserved among species. Proteins belonging to clade 11 present a well conserved string of 42 amino acids (alignment identity of 97%) following the Pfam domains (PF00005) (Additional file 6: Figure S2). Interestingly, a member of this group, At5g39040.1 has been reported as involved in aluminium resistance [32]. Finally, in clade 13 (bootstrap index 70%), which groups 40 ABC proteins, three large expansions were observed in tomato (9 ABCBs), potato (9ABCBs) and rice (10 ABCBs). A perfect conservation of orthologous pairs between tomato and potato on chromosomes 11, 6, 12, 3 and 2 [29,33] has been detected.

#### The ABCC subfamily

ABCC (Additional file 7: Figure S3) is a large subfamily of “full-size”, “forward-orientation” proteins. The phylogenetic tree obtained by comparing 109 proteins displays that three *S. cerevisiae* and six *V. carteri* proteins, and one protein from *O. sativa* (Os04g33700.1) cluster separately. Two distinctive angiosperm clades can be evidenced (bootstrap index >75). Proteins belonging to this subfamily have been found to be involved in cellular processes such as vacuolar transport, detoxification and regulation of guard cell plasma membrane ion channels. Clade 1 encodes 12 proteins, of which four annotated in Arabidopsis (AT1G30400.1, AT1G30410.1, AT1G30420.1 and AT2G34660.1) are involved in detoxification, vacuolar transport of abscisic acid and glucosyl ester, organic anion transport, chlorophyll degradation and modulation of seed phytate content [29,34,35]. A unique orthologous in potato and rice, two members in tomato and four members in grape that are putatively involved in fruit maturation process have been found [36,37]. In clade 2 we underlined four remarkable clusters. In particular, cluster 1 groups orthologous genes of AT2G07680.1 involved in vacuole traffic [38] and cluster 2 contains nine proteins with an identity of 60% to AT3G62700.1, involved in vacuolar transport of abscisic acid glucosylester [39,33]. In cluster 4 there is an Arabidopsis ABCC protein (AT1G04120.1), involved in the regulation of anion and calcium channel activities [39], which presents a high sequence similarity (alignment identity of 77%) with other four eudicot proteins. Cluster 4 also contains angiosperm proteins with an average identity of about 75%.

#### The ABCD subfamily

The ABCD phylogeny tree obtained with proteins belonging to all considered phyla (Figure 3), revealed the evolutionary history of this subfamily. *S. cerevisiae* YKL188w peptide is separated from the two main clades and could be designated as the ancestral protein. ABC transporters belonging to this subfamily have been found to play a role in the peroxisome transport [40,41]. Clade 1 includes full-size” proteins (average identity 76%) with “forward orientation”. In this clade is present the Arabidopsis protein AT4G39850.1 involved in a wide range of substrates for peroxisome uptake [42-44]. A similar function could be hypothesized for the homologous peptides (Os01g73530.1, Os05g01700.1, Solyc04g055120.2.1, Sotub04g020700.1.1) detected in tomato, potato and rice. Clade 2 includes “half-size” proteins. The transmembrane domain (400 amino acids) is very well conserved (84% average identity) among proteins belonging to this clade as well as the NBD 1 domain. Interestingly, the two *Solanum* (Solyc12g017420.1.1 and Sotub12g013980.1.1) proteins and the three AT1G54350.1, GSVIVT01036685001 and Os01g11946.1 transporters show a higher identity with NBD motif of green alga Vocar20007372m (about 60% of identity) and Vocar20009192m (about 70%of identity), respectively (Figure 3B).

#### The ABCE subfamily

*ABCE* subfamily (Figure 4), with only ten proteins detected, was found to be the smallest among the subfamilies analysed in this work. The structure of the phylogenetic tree was extremely useful in tracking the evolution of these “trasporters”, also known as RNase L inhibitors (RLI) [45] (Figure 4A). The two ancestral *S. cerevisiae* (YDR091C) and *V. carteri,* (Vocar20004039m) proteins were more similar to *A. thaliana* (AT3G13640.1) and *O. sativa*, (Os02g18180.1) proteins. Only for *Solanum* spp proteins, a small expansion was observed (clade 1 of Figure 4). In this group there was a *V. vinifera* protein (GSVIVT01036876001) that clustered with the Arabidopsis protein AT4G19210.1 which contains N-terminal “ferrodoxin” (4Fe4S-type) motifs and interacts with nucleic acids [11,46,47].

#### The ABCF subfamily

The phylogenetic tree of ABCF subfamily, obtained by comparing 46 proteins, reveals five clades (Figure 5). Proteins belonging to this subfamily have been found to be involved in stress-associated control [48] and seem to have an ancestral origin since they are highly represented both in *V. carteri* and *S. cerevisiae* (Additional file 1: Table S1). *S. cerevisiae* proteins are included in all clusters except for clades 1 and 5. These two clades show an alignment identity of 61%and 66% respectively and include two *V. carteri* (Vocar20008959m and Vocar20013543m) proteins. Interestingly, Arabidopsis proteins present in clades 1 and 5 (AT3G54540.1 and AT5G64840.1) have been found to be involved in root growth and development [17] and a similar role could be predicted for proteins belonging to such clade. Clade 2, with an alignment identity greater than 75%, includes highly conserved proteins in all species analysed*.* Clade 4 comprises six proteins belonging to *S. cerevisiae* and *V. carteri,* with a low alignment identity (42%). Interestingly, three of these proteins (YPL226W, Vocar20002122, Vocar20002123), one in yeast and two in algae, have an additional chromo-domain (IPR023780).

#### The ABCG subfamily

ABCG, the largest plant ABC transporter subfamily, includes two groups according to Sanchez-Fernandez nomenclature: WBCs and PDRs. Members of the ABCG_WBC_ consist of approximately 600–750 amino acid residues [8] and can be involved in the cuticular lipids extrusion [49,50]. ABCG full-size proteins (ABCG_PDR_) have a NBD domain characterized by four “plant PDR signatures” [35]. Many proteins belonging to this subfamily have been found to be involved in resistance to pathogens, antimicrobial terpenoids and auxinic herbicides, and contribute to the transport of signalling molecules or secretion of volatile compounds [51-53].

#### The ABCG_WBC_ group

The ABCG_WBC_ evolutionary analysis, obtained by comparing 219 proteins, shows a high diversification (Additional file 5: Figure S4). Eleven clades that encompass a number of proteins varying from 31 (clade 7) to 4 (clades 5, 7 and 10) have been produced. Clades 1, 2 and 9 encompass 33% of the sequences analysed. A putative progenitor of clades 1 and 2 could be the *S. cerevisiae* protein YCR011C. In the clade 1 is present AT3G55130.1, which appears to be involved in kanamycin resistance when overexpressed in transgenic plants [54]. In clade 2 we found sequences that are highly conserved in eudicot genomes. Clade 3 and 4 encode transporters that could be involved in lipid/sterol homeostasis regulation required for proper vascular development, likewise the AT1G31770.1 and AT4G27420.1 proteins [50,51,55]. Clade 5 groups three sequences similar to AT3G13220.1 (76% identity), which has been found involved in abscisic acid transport [56]. Clade 7 includes a *S. cerevisiae* sequence (YOL75C) that can be ancestral to the diversification that occurs from clade 7 to 11. Clade 8 includes only 8 rice transporters. Clade 9 includes 21 transporters similar to AT1G17840.1 and AT1G51500.1, which are required for export of wax components such as alkanes [57].

#### The ABCG_PDR_ group

**Figure 6:**
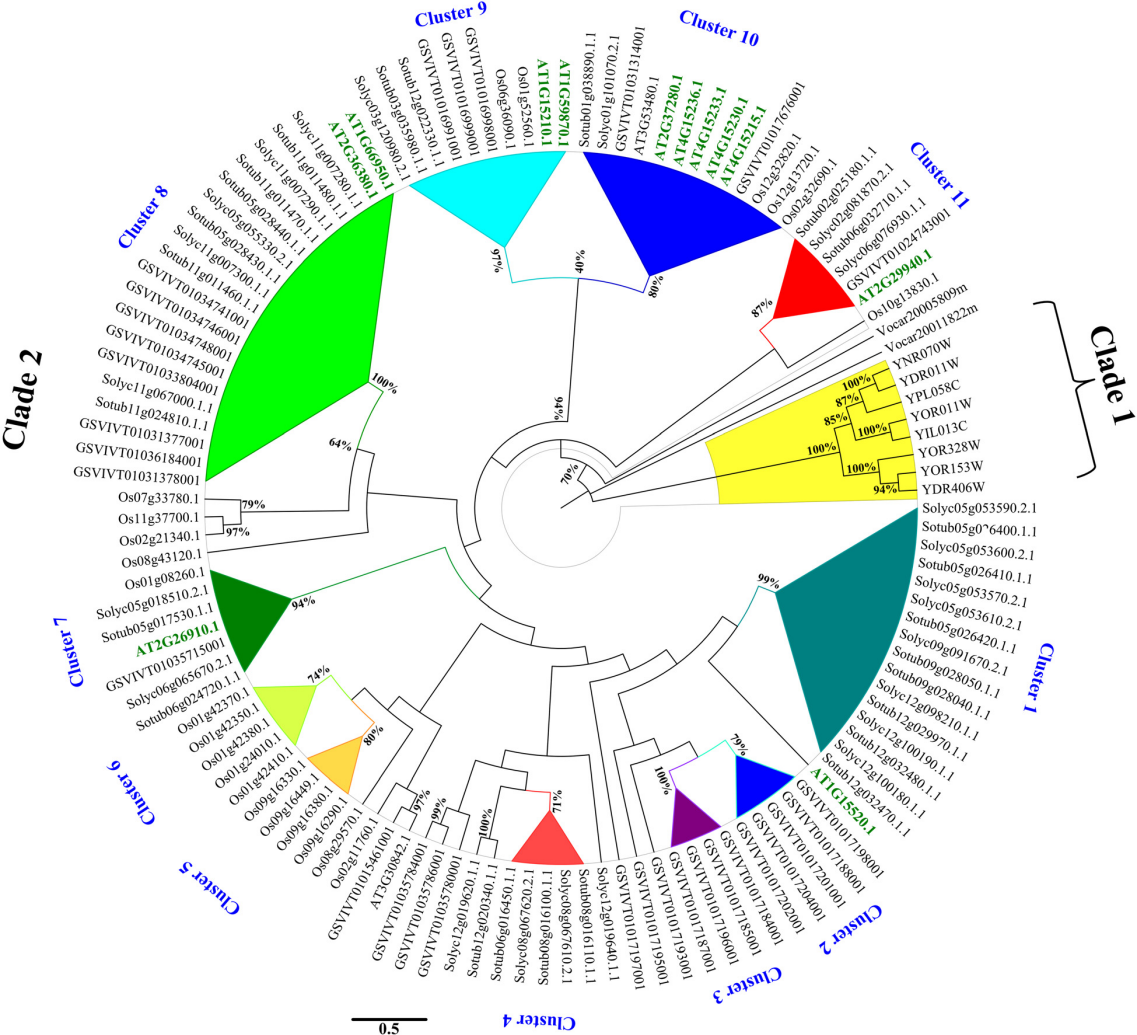
**Phylogenetic tree of PDR group of ATP-binding cassette subfamily G (ABCG**_**PDR**_ **).** Evolutionary analyses were performed as reported for ABCA transporter subfamily.

ABC PDR transporters show a highly conserved PDR domain defined by PFAM database (IPR013581). The phylogenetic analysis, performed by comparing 125 proteins (Figure 6), separated the ABCG_PDR_ proteins in two well definite groups (bootstrap indexes 70 and 100 respectively). Clade 1 (underlined in yellow in Figure 6) includes exclusively 8 *S. cerevisiae* proteins characterized by the PDR domain (PF06422). In yeast, PDR proteins confer resistance to several anti-fungal compounds by actively transporting their substrates out of cell. Next, two *V. carteri* proteins (Vocar20011822 and Vocar20005809) separate clade 1 from clade 2. The sequences of clade 2 are collapsed in eleven clusters marketed with different colours in Figure 6.

Within the cluster 1, 16 *Solanum* proteins are grouped, with a pairwise identity of 82%, located on chromosomes 5, 9 and 12 in tomato and potato genomes. ABCG_PDR_ sequences of this cluster show a high similarity with an ATP-binding cassette transporter (AT1G15520.1) of *A. thaliana* PDR12 (AtPDR12)/ABCG40 known to be involved in pleiotropic drug resistance and abscisic acid (ABA) uptake transport [58,59]. Clusters 2 and 3 are specific for *V. vinifera* while clusters 5 and 6 include only *O. sativa* sequences, suggesting a recent expansion of ABCG_PDR_ in grape and rice. In cluster 7 is present the *A. thaliana*AT2G26910.1 gene, that is involved in cutin formation [35]. The five dicot and monocot orthologous belong to this cluster, could be predicted to be putatively involved in cutin formation as well.

Cluster 8 includes eight *V. vinifera* proteins encoded from genes located on chromosomes 13, 8 and 6 and eleven *Solanum* proteins encoded from genes located on chromosomes 5 and 11, which show a high identity with the *A. thaliana* AT1G66950.1 and AT2G36380.1 genes, known to be highly expressed in the root cells for the secretion of several secondary metabolites [35]. Cluster 9 encompasses an Arabidopsis PDR protein (AtABCG36/AT1G59870.1), which seems to be involved into susceptibility/resistance to the barley powdery mildew pathogen [52]. Eight proteins annotated in tomato, potato, grape and rice with a pairwise identity of 67% also belong to this cluster. Thirteen and six transporters were grouped in clusters 10 and 11, respectively. In cluster 10, the six *A. thaliana* proteins, including AT4G15230 (AtABCG30), a protein involved in root exudation of phytochemicals [35] show an average identity > 60% with the other proteins of this cluster. In cluster 11, a very strong homology (about 75%) among five *Solanum* and *V. vinifera* proteins with A. thaliana AT2G29940.1 protein involved in the modulation of stomata activity [60] was detected.

### Genomic distribution and recent gene duplication events

**Figure 7:**
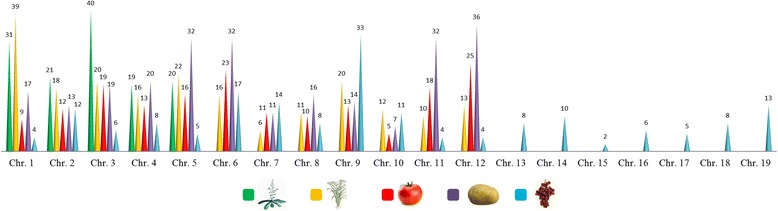
**Distribution of ABC transporter genes across chromosomes for each plant species analysed.**

The genome-wide distribution of Arabidopsis, tomato, potato, rice and grape ABC transporter genes based on the chromosome size was significantly non-random (Arabidopsis p = 0.02; tomato p = 0.005; potato p = 1e-8; rice p = 0.03 and grape =3e-12) (Figure 7). The greatest numbers of ABCs in Arabidopsis were found on chromosomes 3 (about 30% of the annotated genes). In *Solanaceae* genome about 15% of transporters were located on chromosome 12, while the smallest number has been found on chromosome 10. In rice and grape genomes, about 20 and 26% of ABC transporters were positioned on chromosomes 1 and 9 respectively.

Available genomic data provide substantial evidences for abundance of duplicated genes in all surveyed organisms. The gene duplications occupy a leading role in the evolution of genomes. The importance of these events is linked to the necessity of organisms to generate novel functions [61,62]. Detailed computational analysis of individual gene families in different genomic sequences can be used to uncover the mechanisms behind the evolution by gene duplication. In order to discover ABC gene duplications that took place in each analysed genome, we developed a robust system (see Method) for detecting recent duplication events (Additional file 8: Figure S5, Additional file 9: Figure S6, Additional file 10: Figure S7, Additional file 11: Figure S8, Additional file 12: Figure S9, Additional file 13: Figure S10, Additional file 14: Figure S11). A total of 205 ABC genes were involved in recent duplications, with 25% (ranging from 17% to 31%) of annotated transporters in the seven species (Table 2).

**Table 2:** **Identification of recent ABC gene duplication events in all genomes examined**

**Species**	**Tandem duplication**	^**a**^ **Blocks of duplication**	**Total number of duplicate ABCs**
***A. thaliana***	10	4	35^b^ (27)
***O. sativa***	11	2	29 (20)
***S. lycopersicum***	13	2	32 (17)
***S. tuberosum***	19	5	56 (25)
***V. vinifera***	14	6	52 (31)
***V. carteri***	1	-	2 (3)
***S. cerevisiae***	-	-	- (−)
**Total**	67	20	205

^a^A block of duplication was defined if more than one gene was involved in the duplication. ^b^The percentage of duplicate ABC transporter genes on total of gene sequences analysed is reported in brackets.

Overall, the data showed in Table 3 suggest that gene duplication events in vascular plants generated an expansion of some ABC protein families. Probably, this phenomenon is due to the need of a efficient molecular cell interconnection among and within tissues of vascular plants [63].

**Table 3:** **Classification of recent ABC transporter gene duplication events**

**Species**	**n. ABC genes**	^**a**^ **ABC-A**	**ABC-B**	**ABC-C**	**ABC-D**	**ABC-E**	**ABC-F**	**ABC-G**	**ABC-I**
***A. thaliana***	35	5	11	6	-	-	2	9	2
***O. sativa***	29	2	8	4	-	-	-	15	-
***S. lycopersicum***	29	2	8	2	-	-	-	20	-
***S. tuberosum***	56	-	13	9	-	-	-	34	-
***V. vinifera***	52	-	4	14	-	-	2	32	-
***V. carteri***	2	-	-	-	-	-	2	-	-
**Total**	**205**	**9**	**44**	**35**	**-**	**-**	**6**	**110**	**2**

^a^Number of duplicate ABC transporter genes for each considered subfamily.

More than 90% of gene duplications found in this study concern B, C and G sub-families. Instead, transporters belonging to subfamilies D and E showed to be highly conserved (Table 3). In particular 110 genes, out of 465 annotated in subfamily G, are involved in duplication events (Table 3), indicating a considerable implication of this ABC subfamily expansion in all the vascular plants analysed here.

**Figure 8:**
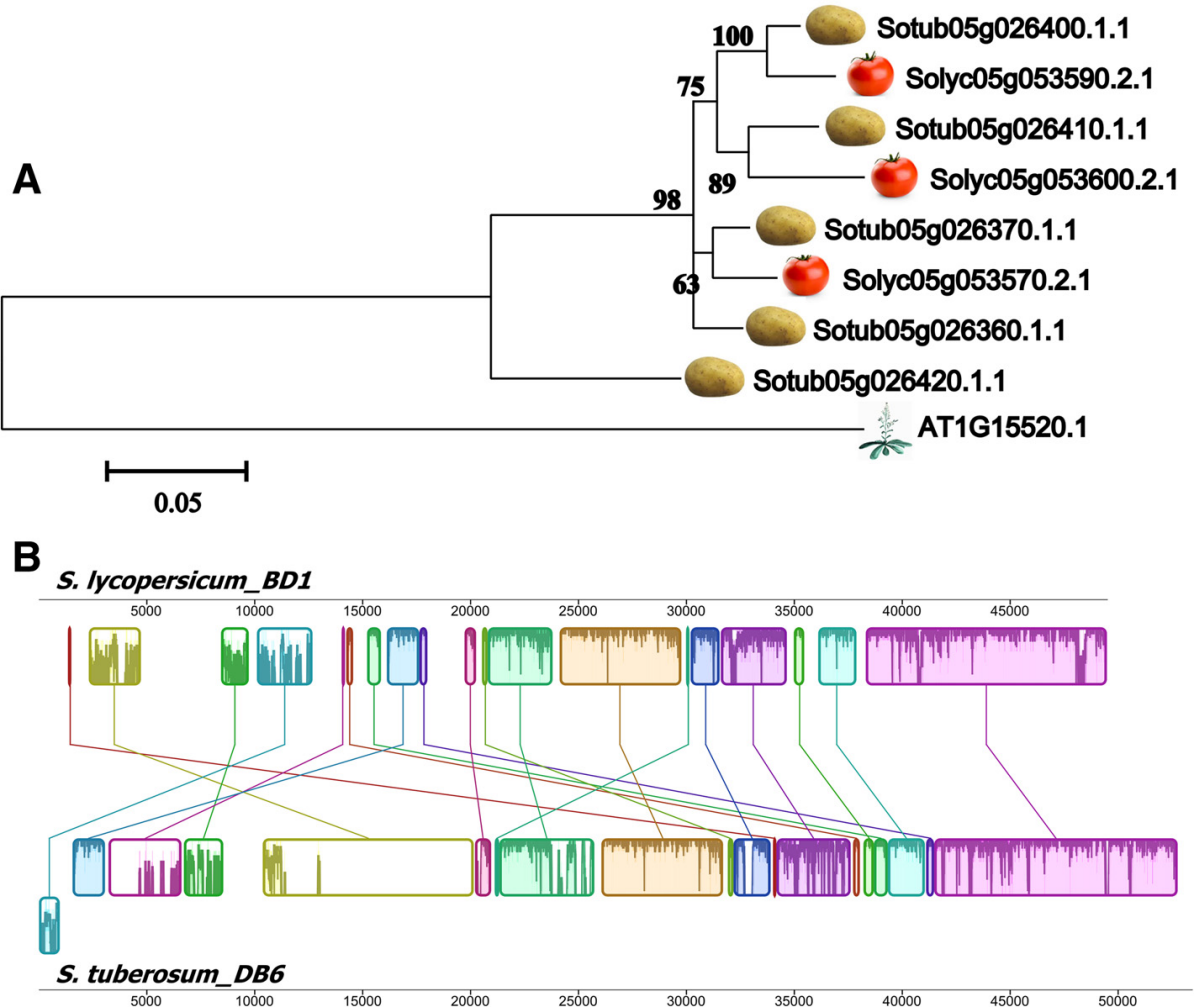
**Locus microsyntenic comparison between** ***S. lycopersicum*** **and** ***S. tuberosum*** **. A)** The evolutionary history was inferred using the maximum likelihood method based on the general time reversible model in MEGA5. Bootstrap values >60% are indicated above branches. The tree is drawn to scale, with branch lengths measured in terms of the number of substitutions per site. **B)** MAUVE alignments of two genomic fragments of about 50 Kb of *S. lycopersicum* and *S. tuberosum* chromosome 5. Similar locally collinear blocks are labelled with the same colour and connected by fine lines. The boundaries of coloured blocks indicate the breakpoints of genome rearrangements.

The following example illustrated a case study of a *Solanum spp.* ABC transporter locus (Duplication Block 1 in Additional file 11: Figure S8; Solyc05g053570.2.1-Solyc05g053600.2.1) under high evolutionary pressure. This region contains five potato and three tomato transporter genes in respective genome, showing a high homology to AT1G15520.1, (average identity about 65%) which is known to be involved in pleiotropic drug resistance, abscisic acid (ABA) uptake transport and lead resistance during *Pseudomonas* infection [58,59,64,65]. The tomato locus on chromosomes 5 showed an average identity 75% with the orthologous potato locus. The genes found in this *Solanum* locus are grouped in the ABCG_PDR_ phylogenetic tree cluster 1. Figure 8A proposed the phylogenetic reconstruction of recent duplication events and orthologous relationships between tomato and potato ABCG_PDR_ transporters. Moreover, a genomic alignment is showed in panel B where the collinear genome blocks confirmed the high conservation of this genomic region in *Solanum* species.

### Tomato expression profile of ABC transporter families

A tomato genome-wide overview of ABC expression profiles was performed to gain insights into the biological role of ABC proteins in tomato. It is already demonstrated that ABC genes can exert their control via transcription expression and that synteny approach is a powerful tool to identify candidates in this species [66]. We analysed the expression profiles of our ABC tomato annotated genes in five different tissues (bud, flower, leaf, root and fruit), by grouping ABC genes according to their subfamily. Of 180 ABC transporters annotated in *S. lycopersicum* Heinz 1706, more than 85% are expressed in at least one of the tissues examined, considering a value normalized as transcripts per million (TPM) > 2 (Table 4). All members of A, D, E and F subfamilies and about 85% of transporters of subfamilies C and I are expressed, while 72% and 67% of ABCB and ABCG genes show a TPM value higher than 2 respectively, suggesting that 15-30% of members belonging to these subfamilies could be pseudogenes. Some of these subfamilies (B, C and G) show also a high rate of gene duplication, suggesting that during the diversification, pseudogenization events could be occurred [67].

**Table 4:** **List of ABC transporters expressed in five tissues (bud, flower, leaf, root and fruit) of** ***S. lycopersicum*** **Heinz 1706 subdivided for subfamilies**

**ABC subfamily**	**n. ABCs expressed in bud**	**n. ABCs expressed in flower**	**n. ABCs expressed in leaf**	**n. ABCs expressed in root**	**n. ABCs expressed in fruit**
**A**	6	6	3	9	4
**B**	16	14	17	19	14
**C**	15	14	22	20	19
**D**	2	2	2	2	2
**E**	2	2	1	2	2
**F**	6	5	5	6	6
**G**_**WBC**_	40	29	25	25	19
**G**_**PDR**_	12	10	15	13	11
**I**	17	18	18	18	18
**Total**	**116**	**100**	**108**	**114**	**95**

**Figure 9:**
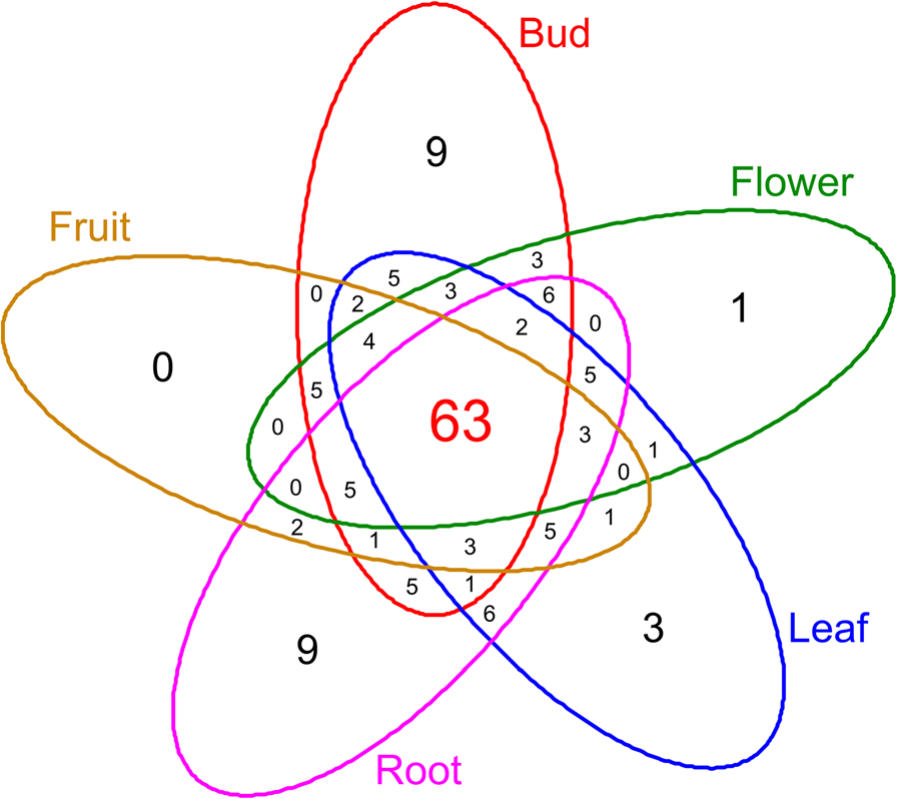
**Venn diagram of tomato expressed ABC genes annotated in root, leaf, bud, flower and 3cm_fruit tissues.**

In Figure 9 a diagram of Venn shows the expression profile intersections of the five tissues analysed, evidencing ABCs expressed in specific tissues of tomato. ABCG_WBC_ (Solyc07g053300.1.1) is expressed only in flower and is located in clade 2 of ABCG_WBC_ phylogenetic tree close to AT1G53270.1 (Additional file 5: Figure S2). Two ABCC (Solyc00g283010.1.1and Solyc11g065710.1.1) and an ABCG_PDR_ (Solyc12g019620.1.1), located on chromosome 0, 11 and 12 respectively, are expressed only in leaf. Nine transporters (Solyc03g113690.1.1, Solyc06g036490.1.1, Solyc06g072090.1.1, Solyc07g065770.2.1, Solyc07g065780.1.1, Solyc09g042280.1.1, Solyc09g042300.1.1, Solyc11g065360.1.1, Solyc12g013630.1.1) are expressed specifically in bud tissues: 8 are ABCG_WBC_ members and only Solyc06g036490.1.1 is a member of the C subfamily. Further, nine genes (Solyc03g093650.2.1, Solyc03g113070.2.1, Solyc03g113080.2.1, Solyc05g051540.1.1, Solyc07g018130.1.1, Solyc08g067610.2.1, Solyc11g067300.1.1, Solyc12g019640.1.1) are specifically expressed in root. About 40% (63) of ABC transporters are expressed in all the five tissues. The complexity of ABC subfamilies expression profiles is showed in nine heat-maps (Additional file 15: Figure S12). Genes with expression profiles characterized by high levels of transcription are surrounded in green. Blue boxes indicate groups of ABC transporters with low levels of expression and include subfamilies C, B, G_WBC_ and I. ABCG_PDR_ subfamily members Solyc05g0553302.1 and Solyc11g0670001.1 are found to be highly expressed in root, confirming the high level of activation reported for their homologues AT1G66950.1 and AT2G36380.1 (see paragraph *The ABC****G***_*PDR*_*group*). Solyc05g0185102.1 is high expressed in fruit and flower and Solyc06g0656702.1 in bud and flowers similarly to the Arabidopsis homologue (AT2G26910.1) found to be involved in cutin formation (see paragraph *The ABC****G***_*PDR*_*group*). The group that shows homology with AT2G26910.1 (see paragraph *The ABC****G***_*PDR*_*group*) has striking differences in terms of expression in specific tissues. Solyc03g1209802.1, homologue to AT1G59870.1 (see paragraph *The ABC****G***_*PDR*_*group*), is highly expressed in fruit, bud and flowers. Solyc01g101070 showed an elevated expression in all analysed tissues. Solyc06g0769301.1, homologue to AT2G29940.1 protein, modulator of stomata activity [55], is highly expressed in all tissues, especially in leaf. Analyzing the expression profile of 32 tomato recent duplicated ABC transporters (Additional file 16: Table S3), high expression level was evidenced for 9 genes in one or more plant tissue. In some case a moderate or comparable expression of other copies belonging to the same duplication block has been observed. Probably, the gene duplication increases promptly the expression level of this gene subfamily in specific tissues [66]. Five duplication blocks showed a very low or level of expression and the duplication block located on chromosome 12 (Solyc12g013630.1.1 -Solyc12g013640.1.1) clearly show the presence of one active copy.

## Conclusion

ABC proteins are firmly established as key players of cellular processes involved in auxin transport, lipid catabolism, xenobiotic detoxification, disease resistance and stomatal function. In this study, 803 ABC transporters were identified by *in silico* analysis of four plant species (*O. sativa, S. lycopersicum, S. tuberosum, V. vinifera*) and 76 transporters in the green alga *V. carteri*, by comparing them with those reannotated in Arabidopsis (*A. thaliana*) and the yeast *S. cerevisiae*. The characterization of ABC proteins based on domain annotation allowed the discovering of new subfamily members. Moreover, we ascertained that ABCG represents the largest group of ABC proteins in all plant species analysed. Phylogenetic analysis allowed us to trace the evolutionary history of plant ABCs, evidencing eukarya diversification. It is well known that a large genome datasets accelerate gene discovery in plants. By analysing the expression data of all tomato ABCs identified in this study, we were able to provide an indication of the putative role of these genes. The results from this work offer useful inputs that may help, for instance, to discover ABC genes with broader or more specific roles, and help to address several biological questions concerning the evolution of the relationships between genomes of different species.

## Methods

### Genomes search for ABC transporters identification

*Oryza sativa*, *Vitis vinifera* and *Volvox carteri* genome data were downloaded from website Phytozome portal [68]. *Arabidopsis thaliana* data were obtained from TAIR database [69] resource. Tomato and potato sequences were provided by the Tomato Genome Sequencing Consortium [70]. *Saccharomyces cerevisiae* strain S288C data were taken from Saccharomyces Genome Database [71]. A BLASTp analysis (e-value < 1e-6) to identify potential ABC transporters in different species were performed [72] (using the entire proteome of each analysed species, starting from 132 ABC protein sequences annotated in *A. thaliana* previously described [8,10].

### Functional prediction of ABC transporters

The set of proteins identified via BLASTp search was further scrutinized using InterProScan software to verify the presence of conserved domains and motifs characteristic of ABC proteins (NBD-TMD). The presence of conserved domains and motifs characteristic of ABC subfamilies (NBD-TMD) allowed us to sort ABC proteins into eight major plant subfamilies (A–I, except subfamily H, which hasn’t members in plants). In this analysis, recovered sequences were compared with the following databases: HMMPanther (Hidden Markov model Panther) to find the characteristic domains for ABC subfamilies, HMMTigr (Hidden Markov model Tigr), patternScan, FPrintScan, HMMPIR, ProfileScan, HAMAP (High-quality Automated and Manual Annotation of Microbial Proteomes), SignalPHMM PROSITE to identify ABC transporters conserved sequences, SuperFamily PRINTS (Fingerprint database), HMMPfam (Protein family) to find “ABC domains”, BlastProDom (Blast protein domain database), and HMMSMART protein motif analyses (Simple Modular Architecture Research Tool, [73] to find ATPase domains. The TMHMM database was also accessed to verify the presence of transmembrane-regions.

### Phylogenetic relationship

Evolutionary analyses of all subfamilies, except for I (Dataset B), were conducted using MEGA5 [74]. The protein sequences were aligned using ClustalW default parameters (v. 1.74) [75]. The phylogenetic relationships were inferred separately for each ABC subfamily using the Maximum Likelihood method. The best phylogenetic method and evolutionary model was determined among candidate models of protein evolution. Models with the lowest BIC scores (Bayesian Information Criterion) are considered to describe the better substitution pattern. For each model, AICc value (Akaike Information Criterion, corrected), Maximum Likelihood value (lnL), and the number of parameters (including branch lengths) are also presented [75]. The bootstrap consensus tree inferred from 100 replicates was taken to represent the evolutionary history of the sequences analysed [76]. The trees were drawn to scale, with branch lengths measured in terms of number of substitutions per site. We have considered significant clades those that have a bootstrap value not less than ≥ 70, containing at least 4 ABC transporter sequences. ABC protein subgroups described in more detail were labelled as “clusters”.

### Recent duplication events of ABC transporter genes

To identify duplicated ABC transporter pairs, we run a phylogenetic analysis using ABC nucleotide sequences of Dataset B, using Maximum Likelihood method and General Time Reversible model.

We defined a gene duplication according to the following criteria: (1) the clade bootstrap index >80, (2) the alignable nucleotide sequence identity ≥70% (3) putative recent duplications were also filtered for physical chromosome co-localization and (4) only one event of duplication is counted for tightly linked genes.

### Evolution rates at codon sites

Selective pressure acting on the ABC-subfamilies were investigated by determining the nonsynonymous to synonymous nucleotide substitution (d_N_-d_S_) indicated as δ. Tests were conducted to estimate the evolution of each codon: positive (d_N_ > d_S_); neutral (d_N_ = d_S_); and negative (d_N_ < d_S_). The variance of the difference was computed using the bootstrap method (1000 replicates). Analyses were conducted using the Nei-Gojobori method [20]. All positions with less than 80% site coverage were eliminated. All the ABC coding DNA sequences were aligned using ClustalW 1.74 [77]. Evolutionary analyses were conducted in MEGA5 [74]. To clearly depict the proportion of sites under selection, an evolutionary fingerprint analysis was carried out using the SLAC algorithm implemented in the Datamonkey server [78].

### Expression data visualization

The expression data of tomato ABC transporters extracted from dataset of the Tomato Genome Consortium [70] were processed as reads per kilobase of the exon model per million mapped reads (RPKM), and subsequently normalized with TPM and visualized with R software [79].

### Availability of supporting data

The data sets supporting the results of this article can be found as Additional files.

## Additional files

Additional file 1: Table S1. ABC proteins identified and classified. The nomenclature of proteins within each subfamily is listed under the Human Genome Organization (HUGO) nomenclature. Additional file 2: Table S2. List of annotated ATP-binding cassette transporter proteins. Additional file 3:
**Sequences of ABC transporters annotated in FASTA format.** All identified of ABC transporter members are included in this file. Additional file 4: Figure S1. Phylogenetic tree of ABCB proteins. Additional file 5: Figure S2. Phylogenetic tree of ABCG_WBC_ proteins. Additional file 6: Figure S3. High conserved amino acidic region among members of ABCB phylogenetic analysis (clade 11). Additional file 7: Figure S4. Phylogenetic tree of ABC-C proteins. Additional file 8: Figure S5. Reconstruction of ABC gene duplication events in *Arabidopsis thaliana*. Additional file 9: Figure S6. Reconstruction of ABC gene duplication events in *Oryza sativa*. Additional file 10: Figure S7. Reconstruction of ABC gene duplication events in *Saccharomyces cerevisiae*. Additional file 11: Figure S8. Reconstruction of ABC gene duplication events in *Solanum lycopersicum*. Additional file 12: Figure S9. Reconstruction of ABC gene duplication events in *Solanum tuberosum*. Additional file 13: Figure S10. Reconstruction of ABC gene duplication events in *Volvox carteri*. Additional file 14: Figure S11. Reconstruction of ABC gene duplication events in *Vitis vinifera*. Additional file 15: Figure S12. Expression profiles of tomato ABCs. Heat map of RNA-seq expression data from root, leaf, bud, flower and 3cm_fruit. The expression values are measured as reads per kilobase of the exon model per million mapped reads (RPKM), and subsequently normalized with TPM (transcripts per million). The colour key indicates the level of gene expression, from red (few reads) to yellow (many reads). Additional file 16: Table S3. Expression levels of tomato ABC genes involved in recent duplication events. For each gene ID are reported: phylogenetic clade name of gene duplication event (GDE); expression values (TPM) from root, leaf, bud, flower and 3cm_fruit and ABC subfamily.
