# Supplementary figures and images for: Genetic variability and evolutionary diversification of membrane ABC transporters in plants
Source: BMC Plant Biol. 2015 Feb 13;15:51. doi: 10.1186/s12870-014-0323-2 (PMC4358917; doi:10.1186/s12870-014-0323-2)

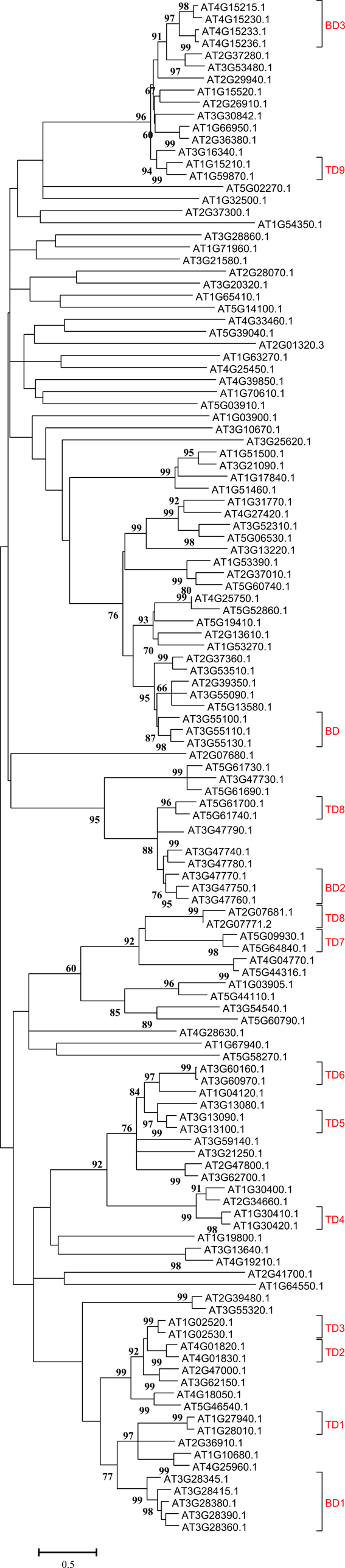

Supplement: Additional file 7: Figure S4. — Phylogenetic tree of ABC-C proteins. [file 12870_2014_323_MOESM7_ESM.png]

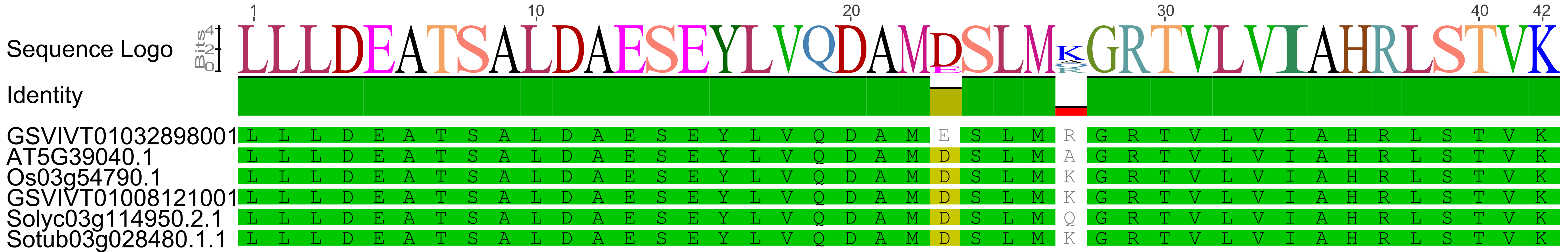

Supplement: Additional file 13: Figure S10. — Reconstruction of ABC gene duplication events in Volvox carteri. [file 12870_2014_323_MOESM13_ESM.png]

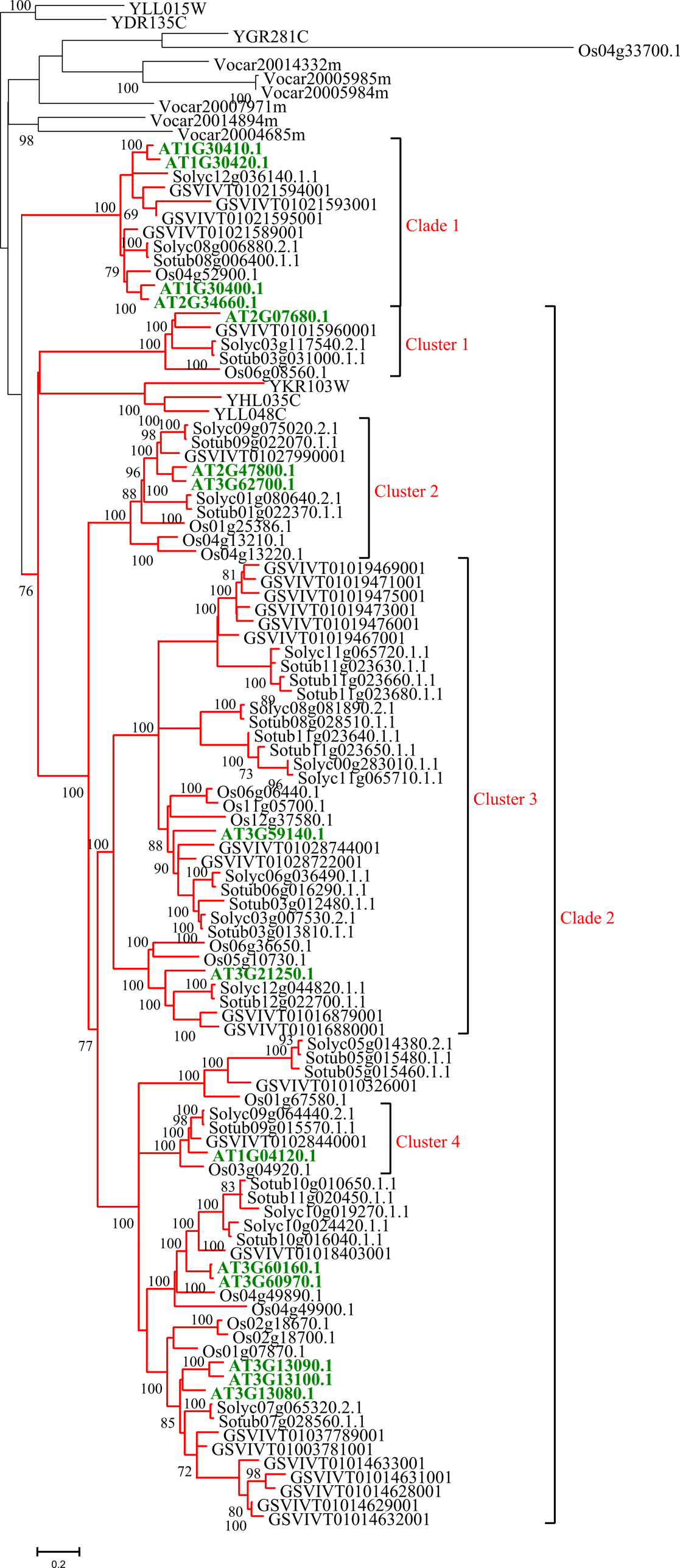

Supplement: Additional file 14: Figure S11. — Reconstruction of ABC gene duplication events in Vitis vinifera. [file 12870_2014_323_MOESM14_ESM.png]
